# Our Pauper Lunatic Asylums

**Published:** 1853-07-01

**Authors:** 


### OUR PAUPER LUNATIC ASYLUMS.

The Act 8 and 9 Vict. c. 126, passed in 1845, has been very generally observed throughout the length and breadth of England and Wales. Only two or three English counties still delay, on some pretext or other, to erect asylums; but as the Commissioners in Lunacy possess ample powers to compel the observance of the act, we feel assured that in a very few years every county in England will have provided a suitable asylum for the detention and treatment of its insane paupers.

We are aware that the act has, in some instances, been unwillingly obeyed; certain counties, chiefly the agricultural, intimidated by the excessive expense which almost invariably attends the erection of any large public building in this country, pleaded poverty and decreasing rates, and wished to defer the providing of a lunatic asylum to more prosperous times. Nevertheless, upon a little friendly persuasion or remonstrance, one county after another set to work to fulfil the requirements of the law, and when once engaged in the task, manifested no further reluctance. The result is a number of public lunatic asylums, which, in architectural design, solidity of construction, comfort, convenience, and adaptation to the use for which they were constructed, certainly surpass those of any other country. We shall presently have occasion to criticise certain points in the arrangements of some of these buildings, but taking them one with another we are glad to speak highly of them, and we consider that nearly all the public asylums recently built confer very great credit on the counties which have erected them, and on the justices who superintended their construction.

The county lunatic asylum now constitutes a remarkable feature in many an English landscape. Commonly placed on a gentle eminence in the neighbourhood of the county-town, it attracts immediate attention by its size, position, and architectural pretensions, and is frequently a very striking and ornamental object.

Foreign travellers in this country are very prone to sneer at the paucity and meanness of our public monuments, but in our county asylums we have a series of edifices, often grand and imposing in themselves, and of higher interest to the philanthropist than any remains of ancient grandeur or mediæval art.

We remarked that the county lunatic asylum is generally a building of considerable architectural pretensions; we may add that, in some instances, it is rather too much so. On making a tour of inspection through the different counties, we find examples of nearly every variety of architectural style; here it is the castellated, there the Elizabethan, next the Italian, and afterwards the county-jail style. In one county the asylum wears the aspect of an union-house, in another it is quite palatial; here it is built on the model of a London hospital, further on it looks like a congregation of suburban almshouses. We do not object to this variety, for we like variety as well as anybody; and we should be sorry to see all our county asylums built on one uniform model; nevertheless, on looking at two or three of the recently-erected buildings, we have regretted that the visiting justices should have extended the non-restraint system to the constructive vagaries of their architects. It has been affirmed that patients are better satisfied to remain in a handsome building than in a mean one, that the grandeur of the place of their detention pleases the fancy, and contributes to reconcile them to their stay: there may be some little truth in this, but we are satisfied there is not much, for we suspect that all the decorative talent of a Barry would fail to render a place of compulsory seclusion agreeable. We would have the asylum spacious, open, airy, and as cheerful as possible, but we would not expend one penny, which might be laid out in providing additional comfort, amusement, or occupation for the inmates, in architectural embellishment or decorative display. We are anxious not to be misunderstood on this head; we wish to see every asylum as handsomely built as the judicious expenditure of the money allotted for its construction will allow, but we protest against wasting any portion of that money in useless ornamental work. A county pauper lunatic asylum is a public edifice designed for charitable purposes; it is paid for by rates which a few contributors administer in the interests of many, and which they are bound to administer economically; it is an eleemosynary institution, and should therefore be plain and simple in its character; it is a refuge for suffering humanity, not a monument raised to the glory of the architect, or the vanity of visiting justices. When we are informed that the cost of building and furnishing the different public asylums in England has varied between 110*l.* and 220*l.* for each patient accommodated; when we find, contrary to all anticipation, that the largest asylums have also been the most costly; we are naturally led to speculate on the causes of this amazing difference, and to wonder why the asylum in one county should have cost just twice as much as the asylum in another.

Although there is this diversity in the original cost of asylums, there is an item in their current expenditure in which they more nearly agree; we refer to the remuneration of the medical officers. Some committees are more generous, we might say more just, than others; but the average salary paid to medical superintendents of public asylums shows how carefully the justices guard the public purse, when mind and not matter is to be paid for. A few thousand pounds for useless battlements, or an inaccessible tower, will pass ungrudged; "*Sir Portcullis Perkins ædificavit, sub auspicio Smithii, Stylesii, Nokesii, Brownii;*" but the salary of the medical superintendent must be reduced to the lowest figure at which there is any chance of obtaining a qualified gentleman to undertake the duties of the office.

For illustration, we will take an institution which the justices concerned in its erection pronounce to be "unrivalled as a Lunatic Asylum, unique in size, elevation, and accommodation, in this country, or perhaps any other" (Report), we mean the Colney-Hatch Asylum,—and certainly the cost of that building should afford something unique. When more than 280,000*l.* has been expended in providing accommodation for less than 1400 patients, the accommodation ought to be of a superior description. We do not profess to know a great deal about the expense of house-building, but we are told that a comfortable six-roomed house may at present be erected for 250*l.*; consequently a village, containing one thousand houses, with church, schools, chapel, market, and cemetery, might be built for the sum which has been spent on this single asylum. We will not pursue the comparison; we have paid our rates, and have got in return a building, which, if not precisely the most convenient, or the best adapted for its uses, is, we will admit, a very handsome and imposing edifice. In selecting Colney-Hatch Asylum as the subject of our remarks, we are not influenced by any peculiar feeling with regard to that establishment; we require an illustration, so we take that asylum which offers the most striking exemplification of those errors in construction and management which it is our intention to indicate and condemn.

The first objection we have to make against the Colney-Hatch Asylum is its extreme size, which far exceeds that of any similar institution in the kingdom. The best and most convenient dimensions for a public lunatic asylum is a question of the highest importance. On inquiring into the opinions of men whose character and experience entitle them to a hearing on this matter, we find them unanimously opposed to the construction of very large buildings. Several foreign physicians engaged in the treatment of insanity, have recorded their disapproval of the extreme magnitude of some of the English asylums. The judgment of the Commissioners in Lunacy is expressed as follows (Report, 1844, p. 23),—"From the best opinions we have been able to collect, and from the result of our own observation and experience, we think it desirable that no asylum for curable lunatics should contain more than 250 patients, and that 200 is, perhaps, as large a number as can be managed with the most benefit, to themselves and the public, in one establishment." With this opinion we entirely concur. In order that an asylum may be efficiently conducted, we consider it absolutely and indispensably requisite that it shall be placed under the supervision of a responsible head. Whether that head is the medical superintendent, or some other responsible officer, does not affect the question; what we have to determine is the limit to the size of an asylum, which renders its effective supervision by the head really practicable. Reflection and observation have taught us that one person cannot usefully direct the treatment of more than 250 patients, and that the charge of that number, demands an amount of labour and anxiety which few men are capable of sustaining. Even in the best conducted asylums a great portion of the actual treatment of the patients is of necessity entrusted to the attendants, and we have found, that although every precaution and discrimination may be exercised in selecting fit persons for this employment, yet only a limited proportion may be implicitly confided in, the remainder requiring constant surveillance to insure the proper performance of their duties. The eye of the master is quite as necessary in an asylum as in any industrial establishment, and no system of divided and delegated authority can compensate for the absence of a responsible chief. We consider the capability of a perfect supervision a very important matter in the construction of asylums, and we think it is not always sufficiently attended to. Take Colney-Hatch, for example: when we look at the plan of that building, and see its widely-spread wings and numerous galleries; when we are told that the united length of the corridors amounts to nearly two-thirds of a mile, we are at a loss to conceive how two medical officers can possibly exercise a satisfactory superintendence over so vast an institution. In short, we regard Colney-Hatch Asylum as a gigantic mistake; and we feel convinced that it would have been better, in every point of view, to have erected three distinct and separate asylums, each capable of accommodating about 500 patients of both sexes, with two resident medical officers, instead of congregating so large a body of lunatics in one overgrown, unmanageable establishment. So far as we know, there is but one plausible argument in favour of large asylums, that is the supposed saving of expense effected in their construction and management. It is stated that an asylum capable of containing 1500 patients can be built and maintained at a much less cost per head than one which will accommodate 500 patients. There is, we admit, a show of reason in this, nay, more, we freely concede the correctness of the principle up to a certain point, for we know that the expenditure of an asylum for 300 patients is, *cæteris paribus*, relatively much below that of an asylum for 100 patients. Nevertheless, we are by no means disposed to conclude that because 300 pauper lunatic patients cost individually less than 100, therefore the relative expense of 1500 and 500 is diminished in the same proportion. But it will be more conclusive to appeal at once to figures. The cost of the care of a pauper lunatic in a county asylum is divided, as most of our readers know, into two heads: 1st, for accommodation, 2nd, for maintenance; the first paid for out of the county rate, the second chargeable to the parochial union to which the patient belongs. The amount of these items varies considerably in the different counties, so that there are not two alike. To obtain a standard of comparison we have calculated the mean cost of building, fitting, and furnishing, including also the price of the land, of twenty county and borough lunatic asylums, in different parts of England, and we find it to be 160*l.* for each patient accommodated. Now Hanwell and Colney-Hatch Asylums, the two largest in this country, have each cost upwards of 200*l.* per patient, that is 20 per cent. above the average—a fact which does not confirm the opinion that large asylums are erected at a lower proportionate expense than smaller institutions. With respect to the cost of maintenance we find, by the respective Reports for 1852, that the average weekly expenditure for each patient was eight shillings and threepence at Hanwell, seven shillings and eightpence at Colney-Hatch, six shillings and sevenpence halfpenny at the Devon, six shillings and fourpence halfpenny at the Somerset, seven shillings and elevenpence at the new Lancashire, and six shillings and twopence halfpenny at the Suffolk Asylums. So it appears that the cost of maintenance of patients at Hanwell and Colney-Hatch is above rather than below the average. But were it a trifle beneath the mean expenditure, we are not disposed to admit that it would show in favour of large asylums, for we believe that the slight saving in the housekeeping department would be balanced by the greater cost of the house service, since in these very large asylums a considerable amount of service is wasted in going about the place. And now, having shown that Colney-Hatch and Hanwell neither cost less to build, nor less to maintain, than smaller asylums, we dismiss the plea of economy commonly advanced in their favour.

But it may perhaps be said, Colney-Hatch Asylum is so complete in all its arrangements, and so superior in every requirement, that it cannot fairly be compared with smaller institutions of infinitely inferior pretensions. Such a remark may have some weight with the general public, but not with those who estimate the comparative merits of the various asylums by the number of cures effected in them. In the last Report of Hanwell Asylum, two pages are filled by a recital of the barbarous treatment to which poor lunatics were formerly subjected; but we all know that these atrocities are now, happily, become matters of history, and that the pauper lunatic asylums are in our times universally conducted with kindness and humanity. This groundwork of excellence is common to all, but beyond this there are some asylums which unquestionably possess peculiar merits, and the best criterion of those merits is the per-centage of recoveries. In the language of the commissioners in lunacy (Report 1844), "the professed, and indeed the main object of a county asylum is, or ought to be, *the cure of insanity.*" It is not merely "a place of refuge," or "a receptacle, or house of detention" for the insane; it is, in its primary intention, a curative establishment; in short, a special hospital for the treatment of a specific disorder. In our estimation, grandeur of elevation, architectural display, and horticultural embellishment, theories of restraint or non-restraint, physic or no physic, are, in the abstract, matters of secondary interest only; the first, the great, the all-important matter, is the curability of insanity, and we regard that system of treatment as the best which effects the greatest number of cures. Looking at asylums in this light, we estimate their comparative merits by the proportion of patients who recover in them. How do Colney-Hatch and Hanwell

Asylums stand by this test? We will take indiscriminately five or six asylums situated in different parts of England, and give the total number of patients treated during the year, with the number of recoveries in each.

| Asylums. | No. treated. | Recoveries. | Per cent. |
|---|---|---|---|
| Somerset, 1852 | 468 | 47 | 10·0 |
| Gloucester, 1851 (paupers) | 380 | 62 | 16·3 |
| Lancaster (Rainhill), 1852 | 545 | 80 | 14·6 |
| Dorset, 1850 | 206 | 28 | 13·5 |
| Kent, 1851 | 682 | 67 | 9·8 |
| Derby, 1852 | 212 | 34 | 16·0 |
| Surrey, 1851 | 1141 | 116 | 10·1 |
| Hanwell, 1852 | 1080 | 43 | 4·0 |
| Colney-Hatch, 1852 | 1628 | 132 | 8·1 |

It is seen by this table, that the total number of recoveries at Hanwell and Colney-Hatch Asylums amounted to 175 out of the 2708 patients treated in 1852, which is 6·46 per cent.; whereas in 1849, the mean per-centage of recoveries in the Cornwall, Lancashire, Oxfordshire, Somersetshire, Suffolk, and Surrey pauper lunatic asylums, was 8·8 of the total number treated in the course of the year.*

Up to this point, then, we have failed to discover any tenable argument in favour of large asylums, and we adhere to our statement, that three separate asylums, each built to contain 500 patients, would be in every respect preferable to any one asylum capable of accommodating 1500 patients.

Having expressed our opinion that the entire active control of an asylum should be confided to a resident responsible head, it may seem almost superfluous for us to state that we think the head ought to be the medical officer. We would have a central legislative board, possessed of supreme power, and exercising a general authority, but we consider that the executive function should be vested wholly in the resident medical superintendent. Viewing a lunatic asylum as a hospital for the treatment and cure of insanity, we hold that all its arrangements, from the plan of the building down to the details of the household economy, should be made subservient to that great end, and we think the medical officer is the fittest person to complete and direct these arrangements. The system of government pursued at many asylums is not in accordance with these ideas, for the visiting justices themselves exercise so general and extensive a control as to make the medical officer merely an agent of their commands.† We have very high respect for any body of gentlemen who sacrifice their ease and leisure to the gratuitous performance of a public duty, but in the case of lunatic asylums, we fear their zeal for the public ser-

* "Psychological Journal," vol. iii. p. 564. There may be circumstances unknown to us which render the pauper lunatics of Middlesex more difficult of cure than the paupers of other counties; if such circumstances exist, they should be mentioned in the Asylum Reports.

† "I cannot conclude the Report without expressing my thanks to the Committee for the interest they have invariably taken in all the troublesome details of the asylum. *No object has been considered too insignificant for their notice,—no circumstance too trivial for their attention.*"—Dr. Hood. "First Report of Colney-Hatch Asylum."

vice sometimes renders them prejudicially active; for without impugning the humanity, intelligence, and business qualifications of county magistrates, we may still question whether they are likely to be so well versed in the general management of a lunatic asylum, and the treatment of insanity, as any well-educated medical man who has long made these matters his particular study. It may perhaps be objected, that although a medical man may possess superior professional knowledge and considerable experience in the treatment of the insane, he may at the same time be wanting in that administrative talent which is indispensably requisite for the efficient direction of so large an establishment as a county asylum; but we are not aware of any circumstance which precludes a member of the medical profession from being as good an administrator as any other gentleman, and the visiting justices, in whom the appointing of the medical superintendent resides, should make inquiries into the business abilities of the respective candidates, and give this point due weight in determining their choice.

This leads us to consider the mode of election of the medical officer of lunatic asylums. The method usually observed is the following:—the justices cause an advertisement to be inserted in the medical journals and in two or three of the leading newspapers, describing the nature of the appointment, and requesting candidates to present testimonials by a certain day. The publicity of the call seems to indicate an open contest, and it is presumable that the best man will stand the fairest chance of success. Many candidates appear; testimonials are obtained, printed, and distributed, the electors are beset with applications; a good deal of money is spent, and a great deal of anxiety excited. By and by the day of election arrives, when, to the surprise and vexation of three or four highly qualified candidates, a gentleman whose qualifications are of the most mediocre description is selected. On inquiry, the rejected candidates learn that the successful competitor is a relative, or connexion, or *protégé* of one of the committee, or that he possessed great private influence or local interest; and they also sometimes learn, with considerable indignation, that the whole affair was a sham, that their testimonials were not examined, nor their claims considered; that the advertising was a matter of form, the choice of the officer having been made before the vacancy was announced. We could name three or four recent elections, of which the above is a true and literal description, and before we proceed, we must protest against all such proceedings; the visiting justices of asylums have the power to elect whom they choose to the post of medical superintendent, but they have no right to advertise for candidates after their choice is virtually fixed, for by so doing they put several gentlemen to considerable trouble and expense, and cause them to waste both time and money. An advertisement published under such circumstances is really little better than an inducement to invest capital on false pretences, and cannot be too strongly reprehended.

But assuming that the justices are sincere in their request, and conscientiously desirous of selecting the best qualified candidate, what are the qualifications which should influence and decide their choice? We think that the requisite qualifications may be divided into three heads. 1, Professional. 2, General. 3, Special qualifications; and on each of these divisions we have a few remarks to offer. Firstly, with regard to professional qualifications, we are of opinion that the possession of any particular diploma should not be too rigidly insisted on. For the last fifteen years the medical profession has been in a transition state; repeated changes have taken place in all the governing medical bodies, and still further changes are advocated and expected, so that many medical men defer entering this or that corporation until matters shall have assumed a more settled aspect. The justices should, of course, demand satisfactory proof that the candidate has received a complete medical and surgical education, but beyond this we see no advantage to be derived from requiring any stated professional degree, for, so far as we know, not one of the governing medical bodies has ever made insanity and its treatment the subject of examination. Secondly, regarding general qualifications; the committee should take into account the age, health, personal activity, moral character, temper, disposition, and business habits of the candidate. They should select a gentleman between thirty and fifty years of age, of good health and sound constitution, of quick and active habits, capable of undertaking a considerable amount of bodily labour, of unimpeachable private character, good-tempered and patient, uniting perfect gentleness of manner with great firmness of will, a tender-hearted, compassionate, and humane man, having feelings consonant with his charitable calling, a good manager, likely to maintain discipline, and able to conduct a large establishment with order and economy. Thirdly, with respect to special qualifications, we mean by this, experience in the management of asylums, and practical acquaintance with the treatment of the insane. The committees of public lunatic asylums would do well to bear in mind, that the treatment of insanity is a distinct and special branch of the practice of physic, that the ordinary routine of medical education does not comprise any instruction in this branch, and that none of the various medical boards ever test a candidate's knowledge of this subject. It consequently follows that the medical man who desires to qualify himself to treat insanity, must make it his particular study; unfortunately, we have no school of psychology in this country; a few lectures were formerly delivered at Hanwell Asylum, and a limited number of medical students were permitted to accompany the physicians in their rounds; moreover, an advertisement appears from time to time in the medical journals, acquainting the profession that lectures on insanity, illustrated by cases, are sometimes delivered at Bethlem Hospital; still we have no school for the systematic study of mental disorders; nor any institution where a medical man can obtain a practical knowledge of the treatment of the insane, and the management of lunatic asylums. At present the only method by which this experience can be acquired is by filling a subordinate post in an asylum. Many of the county asylums, and some of the best private establishments, have an assistant medical officer in addition to the chief, and it is in this corps of assistants that the visiting justices are most likely to meet with a competent superintendent. We consider practical experience the most essential of all the qualifications we have enumerated, yet we know several instances in which no regard has been paid to this most important point, candidates of very limited experience having been selected in preference to gentlemen of many years' practice in this department. In the case of one of the metropolitan asylums, a gentleman who had no practical experience whatever, was selected in preference to several highly qualified applicants, but the successful candidate was fortunate in possessing extraordinary private influence.*

When the visiting justices have elected their medical officer, it remains for them to settle and define the extent of his authority. In our opinion, the entire active administration of the establishment should be vested in his hands, and with it the sole responsibility. In some asylums the resident medical superintendent is styled the director; this appears to us a very appropriate designation, for who is so competent to direct a curative institution as this medical officer? We say to the visiting committee, "Gentlemen, employ all reasonable caution in making your selection; weigh the value of testimonials; inquire well into character and disposition; insist on previous experience; see all the eligible candidates, and examine them in person; and then, when you have appointed the best man, repose full confidence in him, and do not restrict his sphere of action or impair his usefulness by unnecessary regulations or vexatious interference." We have devoted much time and inquiry to a consideration of the relative merits of the different systems of management pursued in various asylums, both in this country and abroad, and we see every reason to believe that the best managed and most serviceable institutions are those of moderate size, in which the resident medical officer is the sole responsible head. In some of our county asylums the medical superintendent is placed in a very derogatory position; he is allotted a mere semblance of authority, and is called upon to share the little he possesses with the steward and the matron. He is a servant where he ought to be the master, and is made responsible for regulations in whose construction he had no voice, and over which he has no effectual control. Now, all this is wrong, and we urge the visiting justices of such asylums to repose more trust in their medical officer, and to confide to him the management of affairs which his professional knowledge and experience render him the best qualified person to conduct.

A very important point, in connexion with lunatic asylums, is the amount of medical supervision, in relation to the number of patients. We repeat, that we consider 250 patients to be the extreme number which ought to be intrusted to the care of one medical officer. The functions of the medical officer of a lunatic asylum are not limited to the ordinary duties of his profession; he has not merely to feel the pulse and look at the tongue, and prescribe physic for his patients, and provide for their personal comfort, but he has to investigate their mental condition, and "minister to the mind diseased." To enable him to do this, he must have time and opportunity to become acquainted with his patients, to

* If challenged, we will relate the particulars of this affair for the amusement and edification of our readers.

gain their confidence, and obtain a personal influence over them. Moreover, as one of the most powerful remedial agents we possess for the cure of insanity is human sympathy, and as that sympathy is the most advantageously employed when established between the sufferer and his medical attendant, the latter should ever strive to win the affections of his patients, and so lead them to regard him as their best and kindest friend, and look up to him as a protector, and even as a parent. We are acquainted with some establishments in which this is the case, and we entertain a firm conviction that the high proportion of cures which distinguishes these very establishments is mainly owing to the confidential and friendly relations subsisting between the patients and their medical director. The majority of the insane are just as conscious of kindness, and quite as susceptible of gratitude, as the generality of mankind at large, and it is in the power of the medical attendant to awaken their gratitude by kindness, and so obtain a standing place in their affections from which to direct the other means of cure. Now this can be effected solely by personal intercourse; consequently, if the number of patients in an asylum is so large as to preclude the possibility of the medical officer becoming personally acquainted with each case, a most valuable and successful remedial agent is lost, and the treatment becomes a matter of routine, instead of being a subject of personal and individual concern. On this ground, therefore, we limit the number of patients to 250 for one medical officer. What then shall we say of the arrangement of Colney-Hatch Asylum, where one medical officer has the sole charge of more than 500 male, the other of more than 700 female patients? We disclaim any intention to disparage the zeal and ability of these gentlemen. We have not the slightest doubt of their performing their multifarious and laborious duties to the full extent of their abilities; but we know the limits of human capacity, and we have had no inconsiderable experience in the management of lunatic asylums, and we defy any mortal man to execute in a satisfactory manner the unconscionable amount of work required of these two unfortunate gentlemen. The medical officer on the female side is reported to have the care, *the exclusive care*, of 730 patients. He is instructed by the regulations to see each of these patients twice daily; he has to make the requisite entries of the admissions and discharges, to collect and to write down the particulars of each case, to devise and direct the moral and medical treatment, to superintend the general management of the asylum, to see that the attendants are well conducted, kind, and attentive to their duties, to settle their misunderstandings and disputes, to investigate and prescribe for all the cases of bodily illness occurring among his patients and his corps of attendants, and after death he has to make a post-mortem examination, and record the morbid appearance. Now, we ask, is it possible for any human being to do all this? Supposing that, one with another, each patient daily requires but a single minute of the medical officer's time, but one minute for all this walking, talking, listening, prescribing, writing, and overlooking, 720 patients would take 720 minutes, that is, precisely twelve hours. We have assigned an average of one minute daily to each individual case, but of course several cases will every day occur, each demanding five, ten, fifteen, or twenty minutes; a case of pneumonia or enteritis could not be examined or prescribed for in less than five minutes. It would require at least ten minutes to extract the essential particulars of a patient's mental state, an additional fifteen minutes to write them out, and a medical man must be unusually expert to be able to perform the post-mortem examination of a body in less than twenty minutes; then there are patients' friends to console and advise, board meetings to assist at, religious worship to attend, professional and scientific visitors to receive, letters to read and answer, the materials of the annual report to collect and arrange, and a host of minor duties which it would be tedious to enumerate. Again, we ask, how is it possible for any medical man, no matter how able and zealous he may be, to perform in a satisfactory manner the enormous amount of labour above specified. And what are the medical officers paid for all this labour? We turn to the report, and find their salaries set down at £200 per annum each. We also find it stated in the same report, that the medical officer of the male department had 732 patients under his care in the course of last year, and his colleague of the female department, 896 patients. So it appears that one of these gentlemen was paid 5*s.* 5½*d.*, the other 4*s.* 5¾*d.* per case. We repeat the fact for the information of the general reader, and for the edification of our professional brethren on the Continent and in America. We repeat that the magistrates for the county of Middlesex, the wealthiest district on the face of the habitable globe, the magistrates who have had the courage to spend nearly 300,000*l.* in building one pauper lunatic asylum, also have the courage to entrust the responsible management of that asylum to two "gentlemen eminently qualified, both by their talents and attainments,"* at salaries of £200 per annum each. But the visiting justices of Colney-Hatch may answer us, by asking why they should increase the salaries of the medical officers, since so many suitable applicants present themselves whenever there is a vacancy. We are fully aware that such is the case, and we have not the slightest doubt that there would be many eligible candidates, even if the salary were reduced to £100 a-year. This, however, does not alter our opinion as to the wisdom and expediency of augmenting the present salaries. We hold it to be an established maxim in industrial economy that the best workmen can be obtained only by paying adequate wages. So with regard to asylums, if the visiting justices wish to attach first-class medical officers, men of science and repute, *permanently* to their institutions, they must give such remuneration as will command their services.

There are many circumstances to induce a member of the medical profession to connect himself for a time with an asylum like Colney-Hatch, were the salary even less than it now is. But we do not imagine that a medical man of any present or prospective eminence will be found willing to remain there for a permanency, at a salary of £200 per annum. The appointment may be held for a time to gather experience, to obtain a professional status, to gain a name, but it offers

* Second Annual Report of Colney Hatch, p. 13.

no intrinsic advantages calculated to attach the holder to the office, and render its prolonged possession desirable. We consider frequent changes in the medical officers of asylums extremely injurious to the utility of such institutions. Yet, in no other asylum in the kingdom do such changes occur so frequently as at Colney-Hatch and Hanwell, and we suspect this is mainly owing to the inadequate salaries. It is by no means creditable to the justices concerned in the management of these vast establishments that the medical officers should be worse paid, relatively to the amount of work performed by them, than the officers of any other county asylum in England. If report speaks truly, it was a short-sighted thrift which deprived Hanwell Asylum of the services of Dr. Conolly; and there are obvious grounds for supposing that that excellent medical officer, Dr. Hitchman, would not have exchanged Hanwell for the Derby Asylum had the salary of the two offices been more nearly commensurate. Two years only have elapsed since the opening of Colney-Hatch Asylum, yet, in that short time both the medical officers have been changed, and it requires no great sagacity to predict that under the system now followed such changes will be of frequent recurrence. If the Middlesex justices are truly desirous to carry out their boast of making Colney-Hatch the "Model Establishment of Europe," they must place the medical staff on a very different footing from what it is at present; they must remember that it is not the magnitude, nor the splendour, nor the costliness, nor the vast expenditure, which confer pre-eminence on such an institution, for these are mere material elements, but it is the spirit which animates, the mind which governs it. The fame of such a building does not consist in the multitude of bricks of which it is constructed, nor in the wide extent of ground it covers, it resides in the names of the illustrious men who are connected with it, the men whose science illuminates, whose humanity hallows it. The first dungeon which Howard visited, the cell in which Pinel first struck off the iron fetters of the maniac, are more famous in the world's history than any other such abodes can hereafter become, even though their "walls were of jasper, their foundations chrysolite." We have but slight hope that our remonstrance will be listened to, we anticipate the curt reply, "Economy, we must study economy—it is for economy that we have one asylum instead of three—that we have no visiting or consulting physician—two resident medical officers instead of six, and that we pay those officers £200 instead of £400 per annum." The plea is a strong one, time-honoured, and of great weight in this country, but why, we ask, should its application be restricted to human intellect and skill when it is not extended to such material things as bricks and mortar, timber and iron? The sum expended in building Colney-Hatch Asylum is proof that no expense was spared to render the construction as complete and perfect as possible, and, although we have criticised the results, we do not question the motives of this enlightened liberality, on the contrary, we only lament that it did not stretch far enough to effect the remuneration of the medical officers. The suppression of the office of visiting physician is, in our estimation, another defect in the management of Hanwell and Colney-Hatch

Asylums. We have been informed that the Middlesex magistrates abolished these offices mainly with the view of saving the very moderate salary that was attached to them.* If such be the case, never was there a more pitiful or less desirable economy. These vast Asylums of Hanwell and Colney-Hatch are not paltry parochial establishments, to be regulated with parochial parsimony, they are public, nay, national institutions, and as such should be rendered, so far as is possible, a public and national benefit. The poor inmates who tenant these charitable institutions have not the power to make any direct or personal return for the expense and trouble of the care bestowed upon them, but they may, unconsciously and with advantage to themselves, be made to aid in the advancement of medical science, and thus reward the assistance afforded them, by increasing our knowledge of the curative treatment of the terrible disorder with which they are afflicted. Before the general foundation of public hospitals how slow was the progress of medicine and surgery; and even these hospitals would have been of little utility without the services of these earnest and inquiring men, by whose industry and talents the sufferings of humanity have been alleviated, and the duration of human life prolonged. Our public hospitals are not only benevolent institutions for the cure of disease, they are also the schools of the healing art, and we most earnestly desire to see Public Lunatic Asylums made subservient to the same purpose. Some of the diseases which afflict mankind are almost wholly confined to the poor and laborious, but insanity is a malady incident to all, it attacks indiscriminately the monarch on his throne and the felon in his cell, and unless arrested will reduce them both to the same extremity of mental degradation. How deeply, therefore, is society interested in the treatment of this malady, and surely no available means should be neglected to enlarge our knowledge of its essential character, and to improve our methods of treating it. Among the measures best calculated to obtain these ends, none seem so likely to be of service as that of attaching men of known and acknowledged eminence in this department of medicine as visiting physicians to our great public asylums. The visiting physician being relieved from the toil and anxiety of superintending the details of treatment, is better able to appreciate results, and has more time and ability to condense, compare, arrange, and classify those results, so as to make them available for the instruction and guidance of others. In France the most talented Psychiatrists of the day are attached to the various public asylums, so that there is hardly an establishment for lunatics in that country which has not at least one practitioner of acknowledged eminence connected with it; why should it not be the same in England?

Another very important regulation in the government of asylums is the position of the matron with respect to the medical officer. We have no hesitation in saying, that it ought to be strictly subordinate, and her functions exercised wholly under his direction. Yet, at Hanwell and Colney-Hatch, and in some other asylums, the matron is not

* This is confirmed by the Hanwell Report for 1852, in which the saving of Dr. Conolly's salary, £315 per annum, is mentioned as calling "for attention and approval," p. 17.

only quite independent of the medical superintendent, but holds the same rank, exercises an equal authority, and obtains the same, or nearly the same, salary! So highly, indeed, do the Hanwell justices esteem their matron, that they print her Report, "replete with interest and pathos, in an unmutilated form," giving it precedence, we surmise, out of gallantry, to the reports of the *medical* officers! We are well aware that many of the matrons of our public asylums are very excellent and very estimable persons, still something more than moral excellence and respectability is required for the successful treatment of insanity, and considering that no matron, in this country, can possibly have received a medical education, and that very few women possess the rare combination of qualities and attainments required for the management of the insane, it seems to us unwise and hazardous to place the matron of the asylum above the control of the medical officer.

Moreover, as moderation in the exercise of authority is not a characteristic of the female mind, this equality of power frequently leads to ill-concealed rivality or more open antagonism, so that the female side of the asylum becomes the seat of a struggle for supremacy, utterly subversive of discipline, and most detrimental to the welfare of the patients. This is no fancy sketch, and we should not have to go out of the county of Middlesex to find a striking example. Another point in connexion with this subject, is the question, whether the wife of the medical officer should in any instance be permitted to fill the place of matron in the same asylum. Our opinion is decidedly opposed to this arrangement. We consider that the matron should be as much under the direction and control of the medical officer as any other servant of the establishment, and our married friends can tell us whether it is probable that such would always be the case if the matron were that officer's wife. Again, it should rest with the medical officer to see that the matron performs her duties, and to reprimand her if she neglects them. Now we fear a conjugal relation betwixt the parties would interfere with the strict observance of this useful regulation. There are other adverse reasons also; some domestic, which we need not particularize; others, respecting which, we may tell our non-professional readers, that most medical men have a strong dislike to their wives knowing anything about the details of their practice. So that altogether we think we have shown sufficient cause why the matron of an asylum should not be the wife of the medical superintendent.

Many of the larger asylums have a resident chaplain, and such as have not, are periodically visited by some neighbouring clergyman. This is an excellent regulation, and one of the great improvements in the modern treatment of the insane. Formerly the poor madman was looked upon not only as civilly, but also as spiritually extinct, so that it never entered the minds of our forefathers that a lunatic could receive consolation from prayer, and participate in the comforts of public worship. But although a large proportion of the insane are fully capable of appreciating the privilege of attending divine service, and derive benefit from doing so, yet there are some patients in every asylum whose delusions forbid any reference to religious subjects, and upon whose condition the indiscreet zeal of the chaplain might produce very injurious consequences. For this reason, we hold that the chaplain should exercise the functions of his sacred office under the direction of the medical superintendent, and that he should be instructed not to address himself particularly to any patient without that officer's knowledge and approval.

There is yet another officer to whom too large a share of authority is conceded in some public asylums; we mean the house steward. It would of course be impracticable for the medical superintendent to see to all the details of the household economy, still we think he should be acquainted with them, and that it should be the duty of the steward to carry them out in accordance with his general instructions; nor would we allow the steward to originate or alter any of the household arrangements, without having previously obtained the sanction of the medical officer.

Lastly, we consider it indispensably requisite for the due maintenance of discipline, that the medical superintendent should possess the full and exclusive authority to engage and dismiss all the attendants and servants. The curative success of a pauper asylum depends in great measure on the character and efficiency of the attendants employed in it; and first-class attendants can be obtained only by paying liberal wages, by treating them well and attending to their personal comforts, and by exercising great care and judgment in their selection. The fixing of the rates of board and wages being part of the general expenditure, falls to the province of the committee; but the selection of attendants should undoubtedly be entrusted to the medical officer, he being the best judge of the peculiar qualities required in this class of persons; and as it necessarily devolves upon him to see that they conduct themselves properly, and perform their respective duties in a satisfactory manner; as they work under his directions, and are immediately responsible to him, they should therefore be placed wholly under his control. If, as at Colney-Hatch, the medical officer is not allowed to engage or dismiss the servants and attendants, the chief mean of discipline is withheld from him, and he is less able to enforce the prompt and implicit obedience which is desirable in these institutions. Furthermore, when the power of the medical officer is limited to suspending the offender, and reporting the offence, he is placed in the false position of appearing as the accuser in a cause of which he is the most competent judge; and in the event of the committee dismissing his complaint, sustains a serious impairment of influence. It is an established maxim in executive administration, that discipline cannot be effectually maintained by a divided authority, and as the strictest discipline is necessary in public asylums, we consider this maxim perfectly applicable to their management.

It may be objected against our measures that they would throw the active government of the public asylums almost entirely into the hands of their medical officers; we willingly admit this, for it is precisely what we wish, and we have a deep and settled conviction that the public utility of such institutions would be largely augmented by the change. A personal acquaintance with many of the medical superintendents of our public asylums, enables us to declare that there exists among and between them a noble emulation to conduct their respective establishments in the most humane and efficient manner, and that in many instances their efforts would be more successful if not impeded by unwise regulations and injurious interference.

To conclude; it should never be forgotten that the abolition of constant mechanical restraint, the introduction of employment, the use of religious exercises, and all the other practical measures which have ameliorated the condition of the insane, and improved the treatment of insanity, have originated with the medical officers of asylums, and with respect to any further alleviation of this dreadful malady which it may please the Author of all good to bestow, we may reasonably hope that the humble instruments of his beneficence will be chosen from the same active body of philanthropists.

---

